# Reduced Fitness and Elevated Oxidative Stress in the Marine Copepod *Tigriopus japonicus* Exposed to the Toxic Dinoflagellate *Karenia mikimotoi*

**DOI:** 10.3390/antiox11112299

**Published:** 2022-11-21

**Authors:** Hongju Chen, Jing Wang, Yunyun Zhuang, Wenzhuo Yu, Guangxing Liu

**Affiliations:** 1 Key Laboratory of Marine Environment and Ecology, Ministry of Education, Ocean University of China, Qingdao 266100, China; 2 Laboratory for Marine Ecology and Environmental Science Laboratory, Qingdao National Laboratory for Marine Science and Technology, Qingdao 266237, China

**Keywords:** harmful algal bloom, *Karenia mikimotoi*, *Tigriopus japonicus*, oxidative stress, toxicity, fitness

## Abstract

Blooms of the toxic dinoflagellate *Karenia mikimotoi* cause devastation to marine life, including declines of fitness and population recruitment. However, little is known about the effects of them on benthic copepods. Here, we assessed the acute and chronic effects of *K. mikimotoi* on the marine benthic copepod *Tigriopus japonicus*. Results showed that adult females maintained high survival (>85%) throughout 14-d incubation, but time-dependent reduction of survival was detected in the highest *K. mikimotoi* concentration, and nauplii and copepodites were more vulnerable compared to adults. Ingestion of *K. mikimotoi* depressed the grazing of copepods but significantly induced the generation of reactive oxygen species (ROS), total antioxidant capacity, activities of antioxidant enzymes (superoxide dismutase, catalase, and glutathione peroxidase), and acetylcholinesterase. Under sublethal concentrations for two generations, *K. mikimotoi* reduced the fitness of copepods by prolonging development time and decreasing successful development rate, egg production, and the number of clutches. Our findings suggest that the bloom of *K. mikimotoi* may threaten copepod population recruitment, and its adverse effects are associated with oxidative stress.

## 1. Introduction

Harmful algal blooms (HABs) have become a global environmental issue in aquatic ecosystems with increasing frequency, intensity, and geographic distribution [1]. High biomass accumulation, phycotoxin production, and oxygen depletion associated with the proliferation of algae adversely impact marine organisms, ecosystems, aquaculture, and public health [2,3]. Dinoflagellates are responsible for most marine HABs, among which *Karenia mikimotoi* is a worldwide HAB-forming species that has damaged the aquaculture industry and threatened ecosystems for decades [4,5,6]. Unlike most of the toxic dinoflagellates, *K. mikimotoi* seems harmless to humans, since no shellfish poisons such as paralytic shellfish poisoning, diarrheic shellfish poisoning, or brevetoxin produced by other species of the genus *Karenia* have yet been identified [4,6]. However, most *K. mikimotoi* blooms are associated with massive fish or shellfish mortality, and this is commonly considered to be caused by haemolytic and cytotoxic effects [7,8,9,10]. *K. mikimotoi* could generate reactive oxygen species, which is also speculated to be involved in *K. mikimotoi*-induced toxicity [11,12,13], and the effects of *K. mikimotoi* on the antioxidant enzyme activity of algae, fish, and shellfish have been reported [14,15,16,17,18], which is potentially associated with its neurotoxicity [15,16]. Although the exact toxicity mechanism of *K. mikimotoi* remains elusive, multiple studies have shown that its toxicity relies on direct contact with intact algal cells [19,20] and could vary greatly among strains and be target-species-specific [4,11].

In marine ecosystems, copepods are the key linkage in food webs, playing a vital role in transferring energy and material, including toxic substances, from primary producer to higher trophic levels. Previous studies on pelagic copepods have shown that *K. mikimotoi* may cause decline of fitness and population recruitment. Adverse effects include reduction of survival, feeding behavior and rate, motility, egg production, and egg-hatch success (Table 1). Compared to fish, shellfish, and other invertebrates such as rotifer, copepods, particularly the benthic lineages, have received less attention in the ecotoxicity of *K. mikimotoi* [4,21]. Whether this toxic species also causes detrimental effects on benthic copepods is largely unknown. The harpacticoid copepod *Tigriopus japonicus* is a neritic and benthic species that has been extensively used in assessing environmental risk of heavy metal, biocide, microplastics, and ocean acidification. [22,23,24]. Its small size, short life cycle, ease of culture and maintenance, and adaptability to various conditions in the laboratory make *T. japonicus* a suitable model species of ecotoxicology [25]. Despite living in benthic habitats, *T. japonicus* is able to effectively utilize a variety of pelagic microalgae [26,27]. A large proportion of primary production precipitating to the sediment after blooms and vertical migration of *K. mikimotoi* could also expose *T. japonicus* to toxic pelagic algae [28].

In this study, we aim to determine whether feeding of *T. japonicus* with *K. mikimotoi* has negative effects on its fitness through both acute and chronic assays. Survival, feeding, and biochemical parameters related to oxidative stress and neurotransmission in copepods exposed to a series of *K. mikimotoi* concentrations were monitored. Sublethal effects were investigated by assessing development time, successful development rate, egg production, and number of clutches for two generations.

## 2. Materials and Methods

### 2.1. Algal and Copepod Cultures

*K. mikimotoi* (strain C32-HK), originally isolated from the South China Sea, was provided by the Research Center for Red Tide and Marine Biology of Jinan University, Guangzhou, China. The nontoxic control *Isochrysis galbana* was obtained from the Algal Culture Center of the Institute of Oceanology, Chinese Academy of Sciences (Qingdao, China). Both algal strains were cultured in L1 seawater medium [35] at a salinity of 30.0 ± 1.0 and a light intensity of 100 ± 10 μmol·photon·m^−2^·s^−1^. *K. mikimotoi* was maintained at 25.0 ± 1.0 °C under a 14/10 h light/dark regime, and *I. galbana* was maintained at 20.0 ± 1.0 °C under a 12/12 h light/dark regime. Copepods *T. japonicus* were originally collected in rocky intertidal pools on the coast of Qingdao, China and maintained at a temperature of 20.0 ± 1.0 °C, salinity 30.0 ± 1.0, under a 14/10 h light/dark regime, and fed with a mixture of *Platymonas helgolandica* (1.0 × 10^5^ cells·mL^−1^) and *I. galbana* (2.0 × 10^5^ cells·mL^−1^). All seawater used was taken from offshore Shazikou, Qingdao, China and filtered through a 0.45-μm polycarbonate membrane and sterilized.

### 2.2. Survival

A 14-day acute test was conducted using adult females *T. japonicus*. Copepods were fed on *K. mikimotoi* of the exponential phase at the concentrations (cell density) of 10,000, 20,000, 30,000, 40,000, and 50,000 cells·mL^−1^. The series of cell densities was chosen based on the abundance in natural blooms and the results of preliminary experiments in which differential survival rates were observed. *I. galbana,* with a concentration of 2.0 × 10^5^ cells·mL^−1^, was used as control prey. Triplicate 100-mL glass beakers containing 10 adult females and 50 mL test solution were set up for each algal concentration and control. The copepods were incubated at 20 ± 1 °C under a 14/10 h light/dark photoperiod. Every 48 h, mortality was recorded, and the test solutions were renewed with fresh algal prey.

An acute test for nauplii and copepodites was conducted for 4 days. For each algal concentration and control, triplicates were set up. For each replicate, 25 nauplii or 15 copepodites were transferred to one well of a 6-well tissue culture plate. Each well contained 8 mL test solution. These nauplii and copepodites were raised under the same conditions mentioned above. Every 24 h, mortality was recorded and the test solutions renewed with fresh algal prey. Finally, 96-h LC_50_ was determined by fitting to a sigmoidal dose–response curve.

### 2.3. Biochemical Assays

Previous studies indicate that feeding on toxic algae can alter the oxidative status in grazers and disturb their antioxidant system [4,11]. Thus, we assessed the ROS production, total antioxidant capacity (T-AOC), and the activities of three key antioxidant enzymes: superoxide dismutase (SOD), catalase (CAT), and glutathione peroxidase (GPx). SOD, CAT, and GPx comprise the first-line defense antioxidants by dismutating superoxide radicals and breaking down hydrogen peroxides and hydroperoxides to harmless molecules, respectively [36]. They are indispensable in the entire defense strategy and commonly used for oxidative status assessment in various organisms, including copepods [37,38]. Moreover, acetylcholinesterase (AChE), the hydrolyzing enzyme of the neurotransmitter acetylcholine, is also a well-established biomarker of various environmental stressors, including toxic algae [37]. Thus, the activities of these biomarkers in copepods fed on *K. mikimotoi* are of particular concern.

Following the 14-day acute test, biochemical assays were conducted in the surviving adult female copepods of each treatment and control. One hundred active adult females of *T. japonicus* with dual egg sacs were used for biochemical assays for each treatment and homogenized on ice in 100 μL 0.9% ice-cold sodium chloride solution.

A portion of the homogenate was centrifuged at 10,000× *g* for 10 min at 4 °C and the supernatant was collected for reactive oxygen species (ROS) assays. The rest of the homogenate was centrifuged at 825× *g* for 10 min at 4 °C, and the supernatant was collected for biochemical assays of T-AOC, SOD, CAT, GPx, and AChE. ROS and enzyme activity were measured using detection kits following the manufacturer’s instructions (Nanjing Jiancheng Institute of Biological Engineering, Nanjing, China). The level of ROS was indicated by the fluorescence of dichlorofluorescein (DCF), measured by F-4700 fluorescence spectrophotometer (Hitachi, Tokyo, Japan) with a 500 nm excitation wavelength and 525 nm emission wavelength. Micro Plate Reader Model ST-360 (Kehua, Shanghai, China) was used for measuring the enzyme activities and total protein concentrations. The ROS levels and enzyme activities were normalized to total protein concentration. Water-soluble protein concentration (μg·mL^−1^) was measured by the bicinchoninic acid assay (BCA), following the manufacturer’s instructions for microtiter assays (Nanjing Jiancheng Institute of Biological Engineering, Nanjing, China). A standard curve was prepared using six bovine serum albumin (BSA) concentrations ranging from 50 to 2000 μg·mL^−1^.

### 2.4. Feeding

Prior to the experiment, mature and healthy adult females were randomly sorted and starved in sterilized filtered seawater for 24 h. Triplicate 100-mL glass beakers containing 10 adult females and 50 mL test solution with diets were set up for each algal concentration and control (*K. mikimotoi*: 100, 200, 400, 600, 800, 1.0 × 10^3^, 2.0 × 10^3^ cells·mL^−1^; *I. galbana*: 0.3 × 10^5^, 0.7 × 10^5^, 1.4 × 10^5^, 2.1 × 10^5^, 3.0 × 10^5^, 3.7 × 10^5^, 7.4 × 10^5^ cells·mL^−1^). Incubation took place for 24 h under the conditions described above with beakers wrapped in foil. Prey concentrations were determined before and after incubation. Algal suspensions were preserved in 2% neutral Lugol’s solution and cell abundance was determined by counting cells per sample using Sedgewick Rafter counting slides.

Clearance and ingestion rates were determined following Frost (1972) [39]:

(1) $$F=\frac{V}{N}\times \frac{lnC_{t}-lnC_{tf}}{t}$$

(2) $$I=F\times \frac{C_{tf}-C_{0}}{\ln C_{tf}-\ln C_{0}},$$

where *F* is clearance rate (mL·ind^−1^·h^−1^), which refers to the volume of ambient medium filtered by individual copepod per unit time; *I* is the ingestion rate (cells· ind^−1^·h^−1^), representing the number of algal cells filtered by individual copepod per unit time; *V* represents the volume of medium (mL); *N* is the number of copepods in each beaker; *C_t_* indicates the final prey concentration in the control beaker (×10^4^ cells·mL^−1^); *C_tf_* represents the final prey concentration in the grazer beaker (×10^4^ cells·mL^−1^); *C*_0_ represents the initial prey concentration (×10^4^ cells·mL^−1^); and T is feeding time (h). Carbon-based rates were calculated using cell size and estimated carbon content per cell.

The carbon content of *K. mikimotoi* and *I. galbana*, respectively, was calculated according to the Menden-Deuer and Lessard equation [40].

(3) $$C=0.76S^{0.819}$$

(4) $$C=0.228S^{0.899},$$

where *C* represents the carbon content of individual cells (×10^−6^ µg·C·cell^−1^), and *S* represents the volume of individual cells (µm^3^·cell^−1^). Cell size, cell volume, and carbon content for each algal species were listed in Table 2.

The functional response data were fitted to Holling Type III models as follows:

(5) $$I=\frac{I_{\max}C_{0}^{2}}{C_{0}^{2}+K_{m}}$$

(6) $$F=\frac{I_{\max}C_{0}{}^{}}{C_{0}^{2}+K_{m}^{2}{}_{}},$$

where *I*_max_ is the maximum ingestion rate, *K*_m_ is the half-saturation constant, and *C*_0_ is the prey concentration. 

### 2.5. Chronic Test

Larval/juvenile development and adult reproduction were investigated in a chronic test, in which copepods were fed on *I. galbana* (2.0 × 10^5^ cells·mL^−1^) as control and *K. mikimotoi* with sublethal concentrations of 500, 1000, 2000, and 4000 cells·mL^−1^. The culture conditions were the same as those described above. 

Newly hatched nauplii (<24 h after hatch) were transferred to 6-well tissue culture plates and each well contained 10 nauplii and 8 mL test solution. Test solution was renewed every 48 h, whereas the mortality, development stage, and development time (nauplii to copepodite and copepodite to adult) were monitored every day. Dead individuals were removed. Individuals that developed into copepodite were selected for further cultivation. These nauplii are reared under the above conditions until the adult female forms an egg sac. For the second generation (F2), 10 nauplii (F2) produced by an F1 female from each treatment or control were transferred to new 6-well plates and maintained under the same conditions. Triplicates for each treatment or control were set up. Survival, developmental time for the nauplius phase, and developmental time for the copepodite phase were recorded. 

To investigate adult reproduction, healthy and active adult females of *T. japonicus* with dual egg sacs were transferred to 6-well tissue culture plates, with each well containing one individual and 8 mL test solution. For each exposure concentration or control, six replicates were set up and maintained under the same conditions described above for 10 d. The total egg production in 10 days, individual egg production, and the number of clutches were recorded. 

### 2.6. Statistical Analysis

All measurements were carried out in triplicate, and the data are given as mean value ± standard deviation (SD). Statistical analysis was performed using IBM SPSS 21.0 (IBM Corp., Armonk, NY, USA). To calculate 96-LC_50_, dose–response curves were fitted by sigmoidal algorithms using the mean values of each group (*n* = 6). One-way analysis of variance (ANOVA) and Fisher’s Least Significant Difference (LSD) test was used to evaluate the difference among groups. Prior to ANOVA, data were log-transformed to meet the assumption of normality and homogeneity of variance. Two-way ANOVA followed by Tukey’s test for post hoc multiple comparison was performed to test the difference among treatments and generations. Degrees of freedom (df) for each variable and the F ratio (F, the mean square of the variable divided by the mean square of each parameter) were calculated. Correlation analysis was performed using Pearson’s test. In all tests, *p* < 0.05 was considered as significant. Data were plotted using Origin 2017.

## 3. Results

### 3.1. Survival

**Figure 1:**
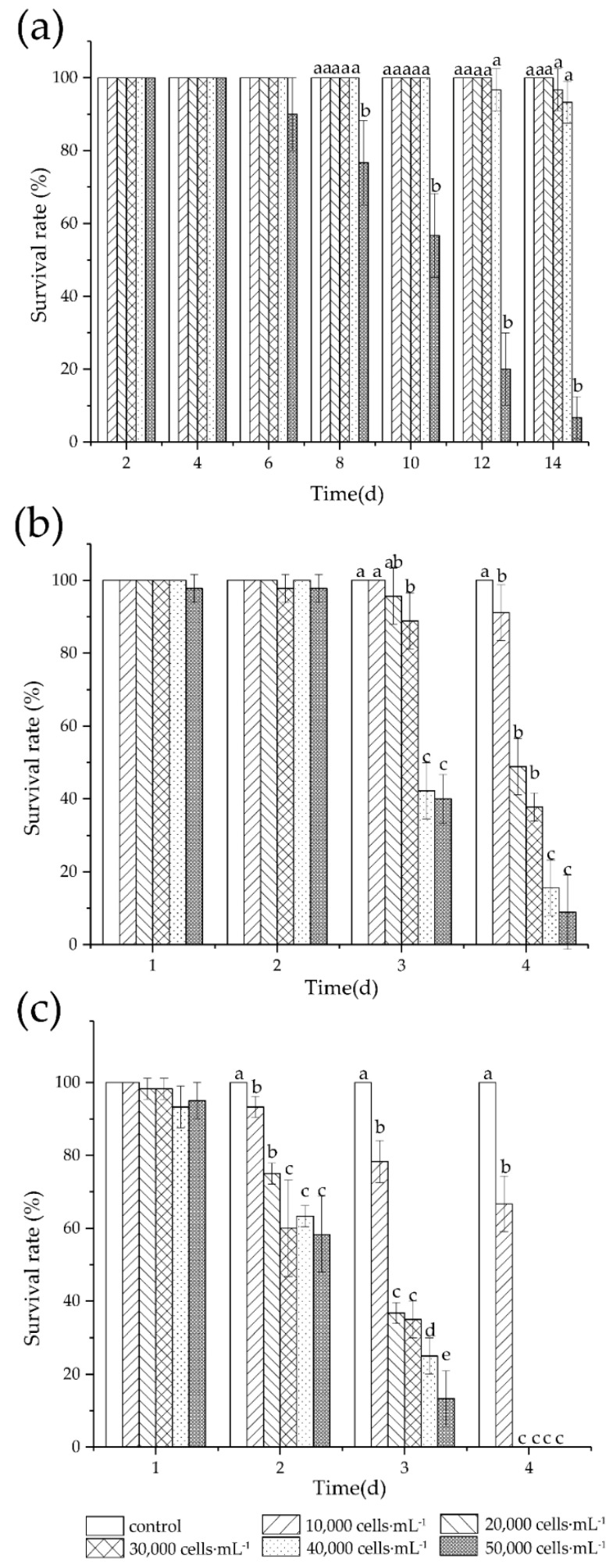
The survival rates of copepod *T. japonicus* adult female (**a**), copepodite (**b**), and nauplii (**c**) exposed to *K. mikimotoi.* (mean ± SD, *n* = 3). Error bars represent SD. Different letters indicate a significant difference among groups at *p* < 0.05.

No mortality of adult female *T. japonicus* was induced by *K. mikimotoi* during the first 4-d incubation, and the survival rates remained high (>85%) throughout the 14-d incubation at concentrations below 50,000 cells·mL^−1^, with no significant difference compared to control (*p* > 0.05; Figure 1). Significant reduction of survival (*p* < 0.05) was only detected at the highest algal concentration (50,000 cells·mL^−1^), and the survival rates decreased with time, reaching 6.67% at day 14. Due to the high tolerance of adult copepod to *K. mikimotoi*, we further assessed the acute effects of *K. mikimotoi* on nauplii and copepodite (Figure 1). Survival rate decreased with time and concentration with 100% mortality of nauplii found at algal concentrations ≥ 20,000 cells·mL^−1^ after 4 days. The 96-LC_50_ of *K. mikimotoi* for nauplii was determined at 10,205 cells·mL^−1^ by dose–response curves fitted by sigmoidal algorithms (*R*^2^ = 0.996, *n* = 6), whereas that for copepodite was about two-fold higher at 21,630 cells·mL^−1^ (*R*^2^ = 0.959, *n* = 6). 

### 3.2. Feeding

**Figure 2:**
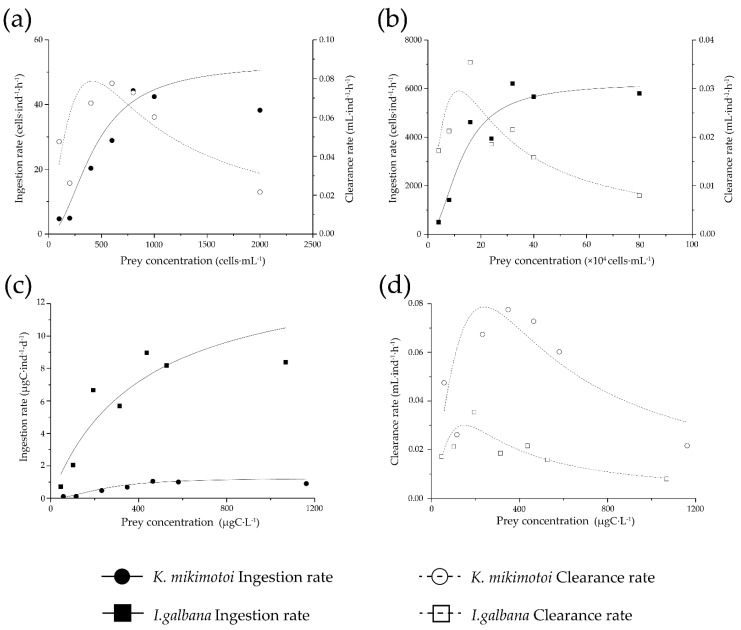
Functional feeding response of adult female *T. japonicus* on *K. mikimotoi* and nontoxic control prey *I. galbana.* Cell-density-based feeding rates of *K. mikimotoi* (**a**) and *I. galbana* (**b**). Carbon-based functional response of ingestion rate (**c**) and clearance rate (**d**). Clearance rates and ingestion rates are presented as a function of the average food concentration. Holling type III equations were fitted with the parameters provided in Table 3.

Concentration-dependent grazing was found on both toxic and non-toxic prey, resembling a typical Holling Type III functional response (Figure 2). The maximum per capita intake of *K. mikimotoi* and *I. galbana* was ca. 44 and 6207 cells per hour, respectively, with half-saturation prey concentration as 430.0 and 127,351.5 cells·mL^−1^. Clearance rate peaked at intermediate prey concentrations. Carbon-based functional responses showed that copepod ingested non-toxic *I. galbana* at a significantly higher rate (*p* < 0.01), whereas copepod had a higher clearance rate on *K. mikimotoi*, which peaked at higher prey concentrations (278 μg C·L^−1^) in comparison with that of control prey *I. galbana*. 

**Table 3:** Maximum ingestion (I_max_) and clearance (F_max_) rates, half-saturation prey concentration (K_m_) estimated by Holling Type III functional response model.

	Clearance Rate		Ingestion Rate					
			Carbon Based			Cell Based		
Prey Species	F_max_ (mL·ind^−1^·h^−1^)	*R* ^2^	I_max_ (ìgC·ind^−1^·d^−1^)	Km (ìgC·L^−1^)	*R* ^2^	I_max_ (cell·ind^−1^·d^−1^)	K_m_ (mL·L^−1^)	*R* ^2^
*K. mikimotoi*	0.078	0.79	1.25	250.32	0.94	52.85	430.95	0.94
*I. galbana*	0.035 ^a^	0.95	8.92	155.08	0.99	6240.17	127,351.46	0.99

^a^ The mean from actual data is provided instead of model-estimated.

### 3.3. Acute Biochemical Responses

**Figure 3:**
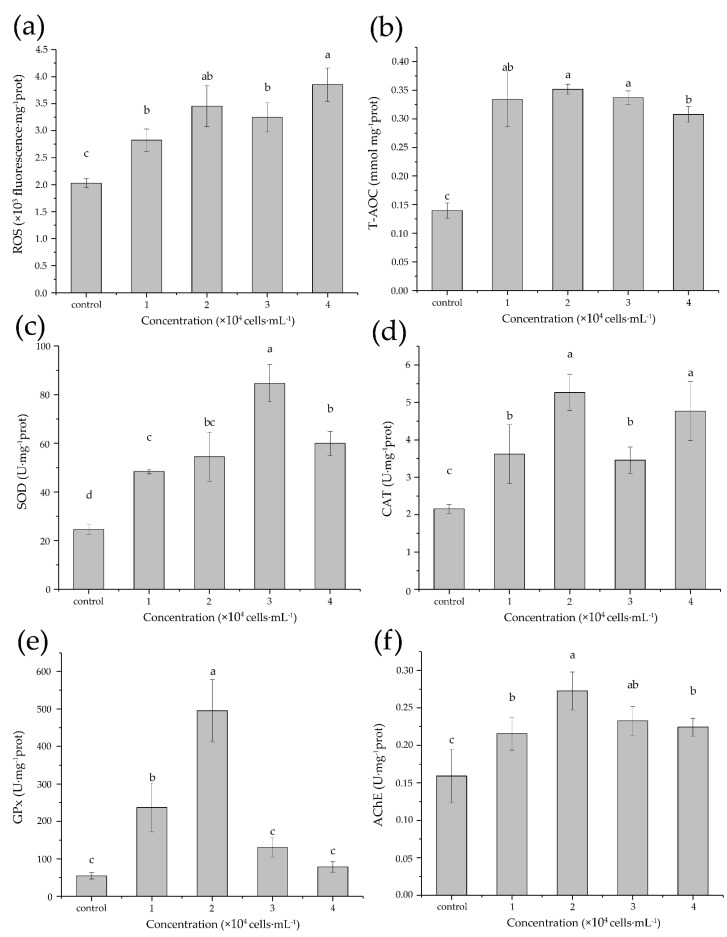
Effects of *K. mikimotoi* on biochemical parameters related to oxidative stress and neurotransmitter in *T. japonicus*. (**a**) reactive oxygen species (ROS), (**b**) total antioxidant capacity (T-AOC), (**c**) superoxide dismutase (SOD), (**d**) catalase (CAT), (**e**) glutathione peroxidase (GPx), and (**f**) acetylcholinesterase (AChE). Different letters indicate a significant difference among groups at *p* < 0.05.

Intracellular generation of ROS in copepods was significantly elevated in all treatments with the highest level (ca. 1.8-fold of control) detected in the copepods fed with 40,000 cells·mL^−1^ *K. mikimotoi*. The T-AOC increased by a factor of 2.2~2.5 in all treatments (Figure 3b) and was positively correlated with the ROS level (*R* = 0.705, *p* < 0.01, Table 4). The cumulative action of antioxidants as indicated by T-AOC was also supported by the stimulated activities of individual antioxidant enzymes, which varied with respect to *K. mikimotoi* concentrations (Figure 3c–e, Table 4). SOD and GPx activity both showed a single-peak pattern that reached 4-fold and 9-fold of the control at 30,000 cells·mL^−1^ and 20,000 cells·mL^−1^ of *K. mikimotoi*, respectively (Figure 3c,e). The peaks were followed by a decrease, even falling back to the level of control in the case of GPx. Exposure to *K. mikimotoi* also noticeably induced CAT activity (1.6-fold to 2.2-fold of control), but the level fluctuated across concentrations (Figure 3d). In the case of AChE activity, significant induction was also found in all treatments. Although the increases (1.3-fold to 1.7-fold of control) were smaller than antioxidants, AChE activity was positively correlated with ROS level, T-AOC, and all antioxidant activities (*R* = 0.679~0.723, *p* < 0.01, Table 4). Furthermore, ANOVA confirmed the influence of *K. mikimotoi* concentration on the biochemical parameters (Table 5). 

### 3.4. Development and Reproduction

**Figure 4:**
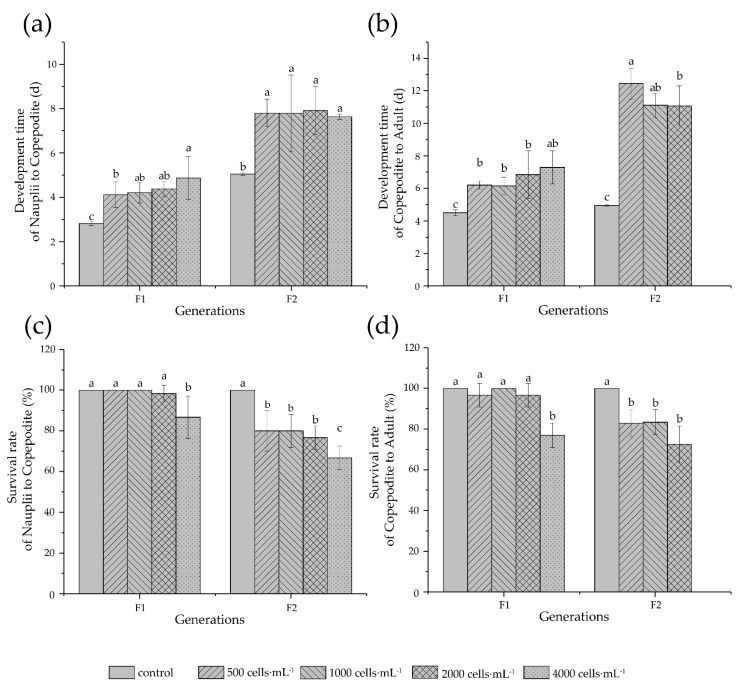
Development time (**a**,**b**) and survival rate (**c**,**d**) in two generations of *T. japonicus* exposed to different *K. mikimotoi* concentrations. (**a**,**c**), nauplius to copepodid stage, (**b**,**d**), copepodid to adult stage. Different letters indicate a significant difference among groups at *p* < 0.05.

A chronic test was conducted at lower exposure concentrations ranging from 500 cells·mL^−1^ to 4000 cells·mL^−1^. Development time and survival were investigated for two consecutive generations. Exposure to *K. mikimotoi* significantly delayed the development of both nauplii and copepodites in F1 but did not significantly affect the survival rate except for the highest concentration (4000 cells·mL^−1^), at which the survival rate decreased to 85% and 80% for nauplii and copepodites, respectively (Figure 4). The adverse influence of *K. mikimotoi* on development was more pronounced in F2. An average of 25.9% of nauplii developed into copepodites in a longer time (8 days) compared to control (5 days), and the development time for copepodite to adult increased by a factor of 2.2–2.5. The survival rate of copepodite decreased to 70–80% at concentrations below 4000 cells·mL^−1^, whereas at 4000 cells·mL^−1^, no copepodite successfully grew up into a adult (100% mortality). Two-way ANOVA (Table 4) further showed the significant impact of *K. mikimotoi* concentration and generation time on the development time and survival rate of *T. japonicus* nauplii and copepodites (*p* < 0.01), and there was a significant interaction between treatment and generation, except for the developmental time of the nauplius stage (*p* < 0.01).

**Figure 5:**
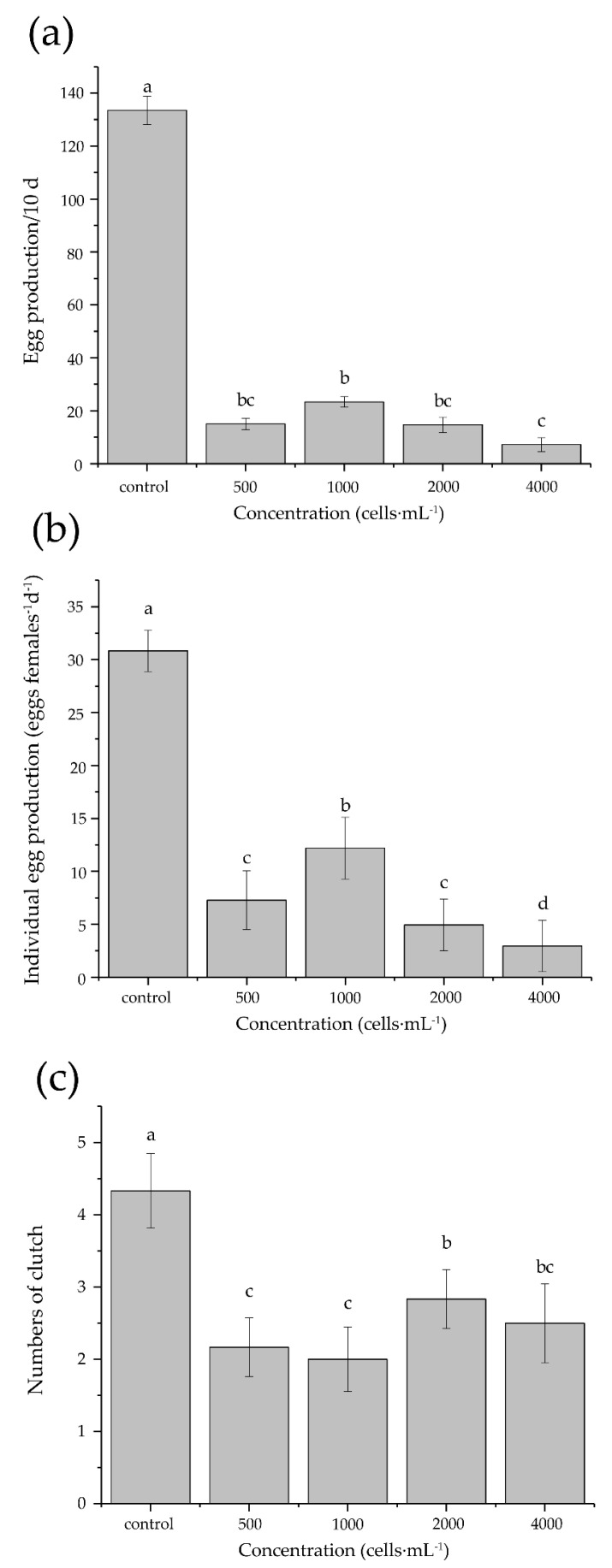
Fecundity (**a**,**b**) and number of clutch (**c**) of *T. japonicus* exposed to different *K. mikimotoi* concentrations. Different letters indicate a significant difference among treatments at *p* < 0.05.

Remarkably, egg production and the number of clutches for F1 were strongly depressed under all tested concentrations of *K. mikimotoi* (Figure 5, Table 6). Total egg production per 10 d and individual egg production reduced to 5.4% and 9.4% of the control, respectively, with the lowest production detected at the highest *K. mikimoti* concentration (4000 cells·mL^−1^). The number of clutches reduced to 46.5–65.1% of the control. No data were available for F2 egg production due to the extremely low number of successfully developed adult females in F2. In addition, deformed and/or detached egg sacs with extremely low egg production were observed in *T. japonicus* exposed to highest *K. mikimotoi* concentration.

## 4. Discussion

### 4.1. Survival

The lethal effect of *K. mikimotoi* has been documented in many marine organisms, such as fish, shellfish, rotifers, and crustaceans, with copepods being one of the less sensitive lineages [19,32]. Within the group of copepods, the sensitivity to *K. mikimotoi* varies among species, and the benthic harpacticoid copepod *T. japonicus* tested in this study seems more tolerant to *K. mikimoti* than the pelagic calanoid copepods [19,29,31,32]. No mortality was observed in adult *T. japonicus* within 96 h at all tested concentrations of *K. mikimotoi*, ranging from 10,000 to 50,000 cells·mL^−1^, and they retained a high survival rate (>90%) during 14-d incubation at concentrations <50,000 cells·mL^−1^ (Figure 1a). Contrastingly, higher mortality has been reported in adult *Pseudodiaptomus marinus* (52–100%, 13 d) and *Calanus sinicus* (20%, 96 h or 22% 16 d) fed with less or comparable concentrations of *K. mikimotoi* [29,31,32]. It has been duly recognized that the toxicity of *K. mikimotoi* is target-species-specific and algal-strain-specific [4,11], which could be attributed to the physiological diversity in structure and defense mechanisms among organisms. Previous studies also postulate that benthic copepods and other benthic invertebrates have a higher degree of tolerance to toxic algae than their pelagic counterparts [27,41,42]. High survival rates are also observed in *T. japonicus* exposed to various environmental stressors [43,44,45]. This is probably due to their physiological acclimation and genetic adaptation to the benthic environments, where the disturbance and contaminant accumulation are high while food supply might be limited relative to pelagic environments.

As expected, nauplii and copepodite *T. japonicus* were more vulnerable compared to adults (Figure 1), which is in concert with findings in *C. sinicus* fed on *K. mikimotoi* [31]. It has been suggested that inadequate feeding due to nonoptimal predator-to-prey size ratio may reduce the survival of nauplii *T. japonicus* [27]. However, the significant concentration and time-dependent decreases of survival in nauplii and copepodite *T. japonicus* revealed in our study indicate that the toxicity of *K. mikimotoi* might be the major factor influencing survival.

### 4.2. Feeding

Both algae were ingested by *T. japonicus* in the concentration-dependent pattern, but the ingestion rate on *K. mikimotoi* was only 5.0–14.3% of that on non-toxic control prey *I. galbana*. The functional response in clearance rate to both prey concentration fit Holling type III, with significantly higher clearance rates found on *K. mikimotoi*. Such depressed ingestion and enhanced clearance on *K. mikimotoi* compared to the control prey are in line with the findings in the pelagic copepod *Temora longicornis* [34]. Reduced grazing on *K. mikimotoi* has also been documented in several copepods, including *Acartia omorii*, *Calanus helgolandicus*, *P. marinus,* and *Pseudocalanus elongatus*, and in several independent studies on *T. longicornis* [29,30,33]. Explanations related to nutritional inadequacy, toxic, or deterrent effect on grazers have been proposed. *K. mikimotoi* lacks essential polyunsaturated fatty acid and amino acid for zooplankton [31], which was observed in copepod *Pseudodiaptomus annandalei* and other marine zooplankton [19]. Alternation of feeding behavior has been convincingly demonstrated by direct observation in several copepods fed on *K. mikimotoi*. Significantly reduced beating frequency of the feeding appendages have been shown for copepod *C. helgolandicus* and *T. longicornis* [30]. Copepods use appendage movements to produce feeding current and further capture food particles; therefore, reduced beating frequency mirrors the lower prey-encounter rate and consequently lower ingestion rate of prey. Moreover, deterrent effects during the process of capture, examination and rejection of viable toxic cells have been observed in copepod *P. elongatus* and *T. longicornis*, and the high rejection rate of *K. mikimotoi* was high compared to that of non-toxic algae [33,34]. These studies argued that the signal molecules produced by toxic algae and remote characterization of the prey by copepod collectively reduce the predation risk, which may account for the long duration of *K. mikimotoi* bloom, as proposed by box-model analysis [46].

### 4.3. Oxidative Stress

Oxidative stress is one of the toxicological consequences of exposure to harmful algae in a variety of aquatic organisms [11,47,48], which refers to the imbalance between pro-oxidant and antioxidant homeostatic cellular conditions or a disruption of redox signaling and control [49]. Such oxidative stress and subsequent damage could be caused by algal-borne ROS, which is also speculated as one of the substances involved in *K. mikimotoi*-induced toxicity [12,13,28], besides hemolytic toxins [7,8,50] and cytotoxins [9,10]. The ROS production of *K. mikimotoi* varies among strains, and some strains such as NGU04 could generate ROS at a level nearly equal to that of raphidophyte *Chattonella marina*, which produces the highest level of superoxide per cell among microalgae [12,51]. However, several studies have suggested that ROS might not play a major role in the toxic effect of *K. mikimotoi*, as shown in rotifers [12,32,52,53].

In spite of this, *K. mikimotoi* could induce significant modulation on ROS production and the antioxidant defense system in aquatic organisms, as many other toxic algae do [47,48,54]. In our study, *K. mikimotoi* stimulated the ROS production in adult *T. japonicus* with a concentration-dependent pattern (Figure 3). ROS production is considered as a metabolic response to toxic algal exposure, which could be induced in minutes to hours [55,56,57], and it even exhibited a high level after a 14-day exposure in our case. Although the mechanism of algae-induced ROS generation remains elusive, recent evidence showed that brevitoxin produced by *K. brevis* inhibits mammalian thioredoxin reductase, a component of the thioredoxin system [58,59,60]. The thioredoxin system is a major cellular antioxidant system that is responsible for maintaining redox homeostasis and is present in all living organisms [61]. Given that cytotoxic polyethers, gymnocin produced by *K. mikimoti*, are structurally analogous to brevetoxins [9,10,62,63], *K. mikimotoi* could potentially disturb the cellular oxidative status in copepod by inhibiting thioredoxin reductase.

Along with the rise in ROS level, *K. mikimotoi* also activated the antioxidant defense system in *T. japonicus*, expressed as enhanced T-AOC, and the activities of SOD, CAT, and GPx (Figure 3), which indicates active ROS scavenging [36]. Oxidative stress indicated by the alteration of antioxidant enzymes is commonly found in *T. japonicus* and other aquatic organisms under various chemical and physical stresses, such as heavy metals, ocean acidification, climate change, and toxic algae [32,64,65]. The effects of *K. mikimotoi* on the antioxidant enzymes have been reported in diatom *Thalassiosira pseudonana* [18], abalone *Haliotis discus hannai* [14,17], zebrafish [15], and medaka *Oryzias melastigma* [16]. Such effects include inhibition or activation followed by inhibition, and the pattern with respect to algal concentration and exposure time varies among enzymes. Although no direct evidence is available, we cannot rule out the possibility that nutritional inadequacy of *K. mikimotoi* may be also a stressor to activate antioxidants in copepods. Here, we used T-AOC to describe the cumulative action of all the antioxidants present in *T. japonicus*, which was significantly induced by *K. mikimotoi* with least variation among concentrations, whereas the activities of SOD, CAT, and GPx fluctuated. In comparison with the control, no inhibition was found in antioxidant enzyme activity, indicating that the oxidative stress imposed by *K. mikimotoi* was still within the antioxidative capacity of *T. japonicus*. However, with the increase of algal concentrations, the elevated enzyme activity of SOD and GPx was followed by a reduction, which points to the possibility that a minimum threshold concentration may be required to depress the enzyme activity. A similar bell-shape pattern has been detected in the SOD activity of abalone exposed to *K. mikimotoi* [14] and several antioxidant biomarkers of zebrafish exposed to microcystins [66]. It has been suggested that activation of antioxidant is energy- and nutrient-demanding [36]. Therefore, exceeding the threshold of concentration or exposure time would exhaust antioxidative capacity and lead to decreased antioxidant levels [67]. Moreover, a positive correlation among antioxidant biomarkers (Table 5) highlighted the cooperation in the antioxidant defense system of *T. japonicus* to counteract *K. mikimotoi* toxicity, in concert with the findings in *T. japonicus* exposed to nickel [38].

Interestingly, a strongly positive correlation was also found between AChE and all oxidative-status-related variables (ROS level and antioxidant activity). We found that with the increase of *K. mikimotoi* concentrations, AChE activity significantly increased, followed by a decrease (Figure 3). Activated AChE activities have been also found in copepods fed on toxic cyanobacteria and several other microcrustaceans under stress [37,67]. AChE has been identified as a biomarker of neurotoxic contaminants in benthic copepods [68]. In concert with previous findings on zebrafish larvae and rotifers [69,70], we observed tetany and hypoactivity in *T. japonicus* during exposure to *K. mikimotoi* (data not shown). Although a neurotoxin has not been identified in *K. mikimotoi*, they produced brevetoxin-like polyethers [9,10,62,63], and genes related to polyketide synthase and saxitoxin synthesis were identified in its transcriptomes [71]. Niu et al. [15] investigated potential *K. mikimotoi* neurotoxicity in zebrafish larvae and found an association between AChE, SOD, and CAT activity and the differential expression of neurodevelopment genes. Whether *K. mikimotoi* exerts neurotoxicity in copepods needs further experimental validation. However, given the remarkable diversity of AChE functions [72], the alternation of AChE activity could be the response to various external stimuli other than neurotransmission, which might be associated with physiological stress, such as oxidative stress [67].

### 4.4. Development and Reproduction

It has been commonly found that toxic algae and their toxins exert adverse effects on the development and reproduction of copepods, but multigeneration toxicity of *K. mikimotoi* in copepods has not been covered in previous studies [29,30,31]. We found that in F1, although *K. mikimotoi* at concentrations < 4000 cells·mL^−1^ could support both nauplii and copepodites of *T. japonicus* to complete their development with high survival, the development time of both stages were significantly prolonged under all tested concentrations (Figure 4). In agreement with a multigeneration study on *T. japonicus* exposed to mercury, the response of F1 at the highest concentration predicted that of future generations [23]. Survival rates of two stages in F1 were significantly reduced at the highest concentration (4000 cells·mL^−1^), and the inhibitory impact of *K. mikimotoi* on development increased in F2, indicating the potential accumulation effect with generations. Remarkably, 70% of F2 nauplii exposed to 4000 cells·mL^−1^ *K. mikimotoi* successfully developed to copepodite, none of which could survive to adult stage. This suggests that copepodite could be more vulnerable under continuous exposure, in accordance with the results of chronic test in *T. japonicus* exposed to the biocide triphenyltin [24]. Contrastingly, our acute test results support the commonly accepted idea that early life stage is more sensitive to environmental stresses, with 96 h-LC_50_ for nauplii being about half of that for copepodite. Such inconsistency may be due to the cumulative damage from repeated or long-term continual exposure, which could ultimately produce more severe effects in the late juvenile stage.

We further found that the exposure to *K. mikimotoi* significantly reduced the egg production and number of clutches in *T. japonicus*, which is in concert with previous reports in the calanoid copepods *A. omorii, C. helgolandicus*, *C. sinicus*, *P. marinus*, and *T. longicornis* [29,30,31]. Although in some cases the hatching success of copepods may not be affected by *K. mikimotoi* [23], and due to the hormesis effect [73], low concentrations of some toxic algae such as cyanobacteria *Nodularia spumigena* and *Colichlodinium polykrikoides* may be beneficial to copepod reproduction and development [74,75], our findings confirmed that *K. mikimotoi* suppressed the reproduction of *T. japonicus*, even at the lowest tested concentration (500 cells·mL^−1^). Copepods exposed to toxic algae for a long period may lack enough energy and nutrients to complete their development and reproduction, due to the high maintenance cost for detoxification [60,76]. The situation may get worse when the algae is nutritionally inadequate, as with *K. mikimotoi* [19,31], and ATP synthesis is inhibited by some environmental stress [77]. Although Wang et al. [27] argued that the egg sac of *T. japonicus* can protect eggs from external environmental disturbances, the detrimental effect of *K. mikimotoi* on the reproduction of benthic copepods is comparable to that on pelagic copepods. Moreover, the number of successfully developed adult females in F2 was too low to conduct subsequent evaluation on egg production, which indicates that *K. mikimotoi* may also affect the sex determination of copepods. Sex determination in copepods is under strong environmental control [78], such as temperature [79], food quality and quantity [80], and toxic algae [81]. In our study, the chronic exposure to *K. mikimotoi* in both parents and offspring may have disturbed the sex determination in F2, and the decreased number of adult females was also observed in F2 *T. japonicus* exposed to biocide [24]. The prolonged development time, the decrease of development success rate, egg production, number of clutches, and female adults in *T. japonicus* collectively demonstrate that population recruitment of copepods could be adversely affected by *K. mikimotoi*, particularly in long-lasting blooms.

## 5. Conclusions

Adult *T. japonicus* showed high tolerance to toxic dinoflagellate *K. mikimotoi* with significant reduction of survival only detected at the highest algal concentration. But nauplii and copepodites were more vulnerable compared to adults, and survival rate decreased with time and concentrations. Exposure to *K. mikimotoi* depressed the grazing of copepods but significantly elevated oxidative stress and the level of AChE. Strong positive correlations among biochemical parameters highlighted the cooperation in the antioxidant defense system of *T. japonicus* to counteract *K. mikimotoi*, and oxidative stress may be associated with other physiological stress induced by *K. mikimotoi*. Moreover, *K. mikimotoi* exerted detrimental effects on development and reproduction in *T. japonicus*. Our findings suggest that the bloom of *K. mikimotoi* may threaten copepod population recruitment, and its adverse effects are associated with oxidative stress.

## Figures and Tables

**Table 1:** Summary of studies revealing the adverse effects of *K. mikimotoi* on copepods.

Copepod	*K. mikimotoi* Strain or Source	Adverse Effects	References
*Acartia omorii*	Japanese coastal waters	Survival, feeding, egg production	[29]
*Calanus helgolandicus*	-	Feeding, egg production	[30]
*C. sinicus*	East China Sea	Survival, reproduction	[31]
*C. sinicus*	East China Sea	Survival	[32]
*Pseudocalanus elongatus*	K-260, Oslofjorden	Feeding	[33]
*Pseudodiaptomus marinus*	Japanese coastal waters	Survival, feeding, egg production	[29]
*Pseudodiaptomus annandalei*	South China Sea	Survival	[19]
*Temora longicornis*	-	Feeding, egg production	[30]
*T. longicornis*	K-260, Oslofjorden, Norway	Feeding	[33,34]
*T. longicornis*	CCMP429, Sutton Harbor, UK	Feeding	[34]
*Tigriopus japonicus*	Coast of Qingdao, China	Survival, feeding, biochemical response, development and reproduction	this study

**Table 2:** Algae characteristic parameters used in the experiment.

Algae	Cell Size (μm)	Cell Volume (μm^3^)	Carbon Content (×10^−6^ µg·C·cell^−1^)
*K. mikimotoi*	26.9	10,146.6	3368.4
*I. galbana*	5.1	35.3	8.05

**Table 4:** Effects of *K. mikimotoi* concentrations on biochemical parameters in *T. japonicus*.

Biochemical Parameter	F	df	*p*
ROS	20.02	4	0.000
T-AOC	39.90	4	0.000
SOD	47.21	4	0.000
CAT	13.79	4	0.000
GPx	40.31	4	0.000
AChE	8.68	4	0.003

**Table 5:** Pearson correlation matrix on biochemical parameters in *T. japonicus* exposed to *K. mikimotoi*.

	ROS	T-AOC	SOD	CAT	GPx	AChE
ROS	1.000					
T-AOC	0.705 **	1.000				
SOD	0.676 **	0.701 **	1.000			
CAT	0.766 **	0.733 **	0.356	1.000	1.000	
GPx	0.256	0.528 *	0.058	0.569 *	1.000	
AChE	0.679 **	0.721 **	0.701 **	0.723 **	0.700 **	1.000

Asterisks indicate the significance of correlation, * *p* < 0.05, ** *p* < 0.01.

**Table 6:** Effects of *K. mikimotoi* concentration and generation on the development in *T. japonicus*.

Response Variable	Treatment			Generation			Treatment × Generation		
	F	df	*p*	F	df	*p*	F	df	*p*
Nauplii to copepodite evelopment time	16.7	4	0	220.6	1	0	3.8	4	0.156
Copepodite to adult development time	43.7	4	0	187.9	1	0	19.9	3	0
Nauplii to copepodite development success rate	19.5	4	0	84.5	1	0	5.5	4	0.002
Copepodite to adult development success rate	16.8	4	0	38.8	1	0	5.3	3	0

## Data Availability

The data presented in this study are available in the article.
